# Cellular Senescence and Senotherapies in the Kidney: Current Evidence and Future Directions

**DOI:** 10.3389/fphar.2020.00755

**Published:** 2020-05-26

**Authors:** Marie Helena Docherty, David P. Baird, Jeremy Hughes, David A. Ferenbach

**Affiliations:** ^1^Department of Renal Medicine, Royal Infirmary of Edinburgh, Edinburgh, United Kingdom; ^2^Centre for Inflammation Research, Queen's Medical Research Institute, University of Edinburgh, Edinburgh, United Kingdom

**Keywords:** senescence, kidney, fibrosis, regeneration, aging, pharmacokinetics, pharmacotherapeutic approaches

## Abstract

Cellular senescence refers to a cellular phenotype characterized by an altered transcriptome, pro-inflammatory secretome, and generally irreversible growth arrest. Acutely senescent cells are widely recognized as performing key physiological functions *in vivo* promoting normal organogenesis, successful wound repair, and cancer defense. In contrast, the accumulation of chronically senescent cells in response to aging, cell stress, genotoxic damage, and other injurious stimuli is increasingly recognized as an important contributor to organ dysfunction, tissue fibrosis, and the more generalized aging phenotype. In this review, we summarize our current knowledge of the role of senescent cells in promoting progressive fibrosis and dysfunction with a particular focus on the kidney and reference to other organ systems. Specific differences between healthy and senescent cells are reviewed along with a summary of several experimental pharmacological approaches to deplete or manipulate senescent cells to preserve organ integrity and function with aging and after injury. Finally, key questions for future research and clinical translation are discussed.

## Introduction

**Figure 1:**
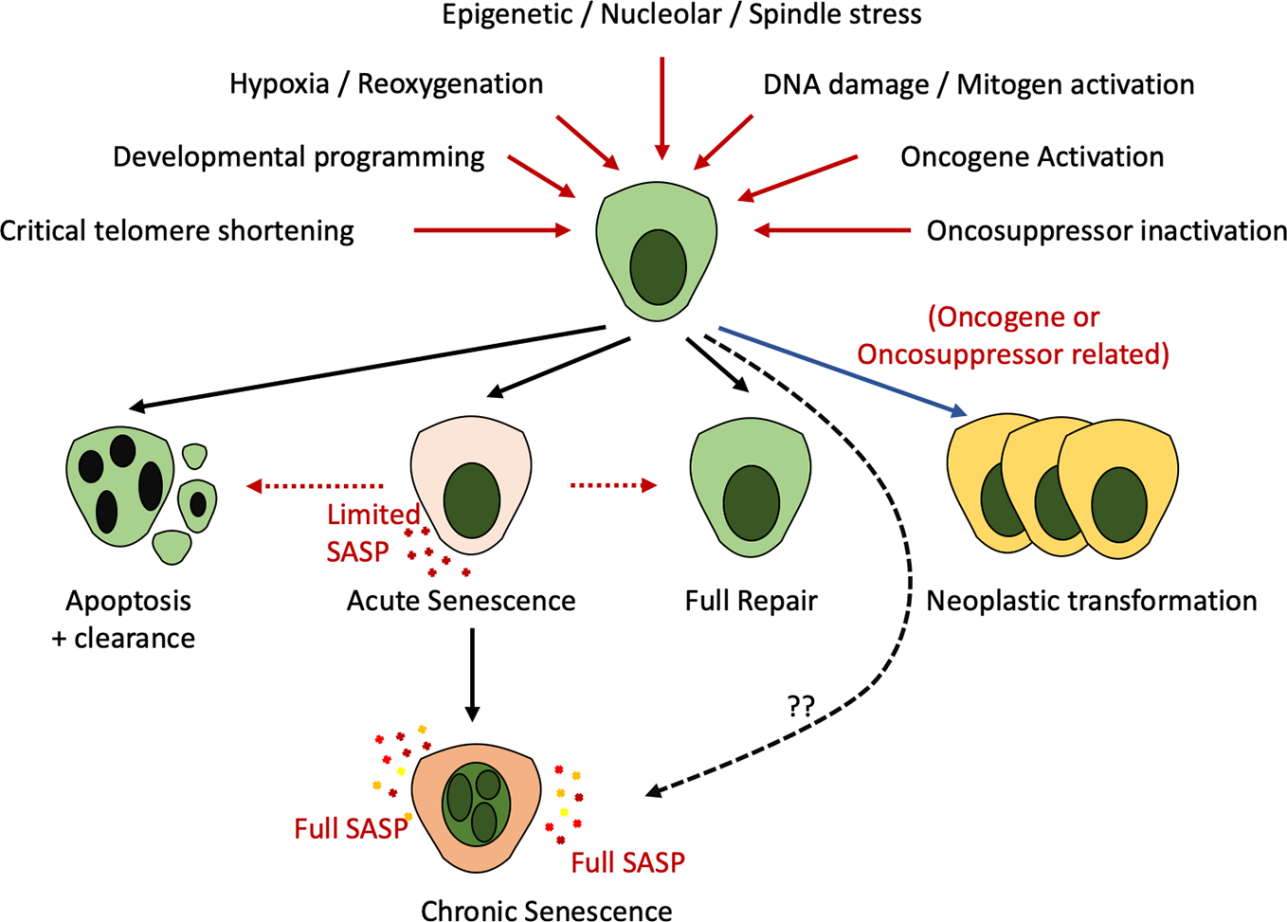
Recognized stimuli to cell fate decisions including apoptosis, senescence, repair, or neoplastic transformation. Multiple developmental, stress-associated, or DNA damage induced cues (red lines) can result in apoptosis, senescence, neoplastic transformation (recognized only in the context of DNA damage—blue line) or full repair. Existing evidence indicates that cells enter “acute” senescence in response to these cues which may lead to repair, clearance, or evolution to the transcriptionally altered “chronic” senescent cell. Whether altered injury cues may generate a cell with immediate properties of “chronic” senescence remains unknown (dotted line).

Cellular senescence is characterized by a typically irreversible cell cycle arrest coupled with widespread alterations at a transcriptional, metabolic and secretory level along with modified cellular morphology and chromatin organization (Sturmlechner et al., 2017; Docherty et al., 2019; Gorgoulis et al., 2019). Senescence is induced as a physiological component of development or in response to multiple insults including genotoxic injury, oncogene activation, cellular stress, mitochondrial dysfunction, lack of nutrients, or hypoxia (**Figure 1**).

The term senescence derives from the Latin “Senex” or “old” and was first used by Hayflick and Moorhead in 1961 following their seminal observation that human diploid cells have a finite capacity to replicate *in vitro* (Hayflick and Moorhead, 1961). Hayflick himself attributed his discovery to aging at the cellular level and the description in their paper is now recognized as replicative senescence occurring due to critical telomere shortening.

The association between aging and senescence is now well established (Campisi, 2013; O'Sullivan et al., 2017), while accumulating evidence has demonstrated that senescent cells also have important physiological and pathophysiological roles in a number of other biological processes including embryonic development (Munoz-Espin et al., 2013; Storer et al., 2013), tumor suppression (Serrano et al., 1997), wound healing (Jun and Lau, 2010), and tissue repair *in vivo* (Krizhanovsky et al., 2008). Of note, recent experiments depleting senescent cells in models of aging *in vivo* have been shown to postpone the onset of age-related diseases and extend healthy lifespan, igniting clinical, and research interest and inspiring the development of targeted senolytic drugs to eliminate senescent cells associated with age and disease (Baker et al., 2011; Baker et al., 2016; Xu et al., 2018).

In this review, we examine our current understanding of the physiological and pathological roles of cellular senescence, with a focus on the kidney and reference to other organ systems where appropriate. We discuss the genetic and pharmacological approaches that have been used to manipulate senescent cell numbers and the potential impact these therapies may have on human health in the future.

## The Influence of Injury Type and Timing on Senescence Outcomes

Cellular senescence is a complex, diverse, and dynamic process. It can be triggered by a wide variety of stressors in many different cell types. There is also accumulating evidence that part of the heterogeneity seen in senescent cells reflects temporal changes in their transcriptome (Hernandez-Segura et al., 2017) and phenotype and resultant influence this has on their environment and clearance patterns (van Deursen, 2014; Herranz and Gil, 2018). Current evidence indicates that chronic senescence evolves from acutely senescent cells in the absence of immune mediated or programmed cell death and clearance.

“Acute senescence” appears to have a physiological role limiting fibrosis in response to injury *via* fibroblast senescence induction, in successful embryonic organogenesis and tissue homeostasis (Krizhanovsky et al., 2008; Jun and Lau, 2010; Munoz-Espin et al., 2013; Demaria et al., 2014). In these tightly controlled processes, the senescent cells appear to be a key component in healthy wounding and are subsequently removed by leukocytes including macrophages and Natural Killer cells in a timely manner (van Deursen, 2014).

**Figure 2:**
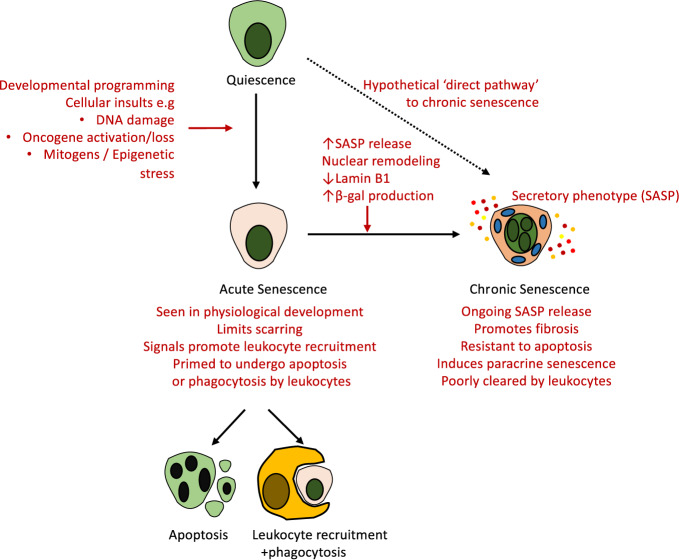
Routes towards clearance or persistence of senescence *in vivo*. Senescent cells can be generated in response to multiple physiological and pathological stimuli. The canonical pathway involves the onset of acute senescence—a state associated with leukocyte recruitment and clearance of senescence—as seen in development and wound healing *in vivo*. With advancing age and in response to other stimuli there is often incomplete clearance of senescent cells, which undergo further phenotypic alterations, upregulate SASP release, nuclear remodeling, and alter the expression of multiple genes including Lamin B1 and β-gal. These chronically senescent cells appear resistant to apoptosis and phagocytosis, and are believed to mediate organ dysfunction and fibrosis *via* their secretory phenotype. Whether these altered outcomes reflect altered initial stimuli, the cell type, the age of the subject, or other unknown factors remain incompletely understood.

In “chronic senescence”, the senescent cells persist and accumulate within affected organs. This can be triggered by a number of insults including critical telomere shortening as a result of repeated cell division (d'Adda di Fagagna et al., 2003), DNA damage (Rodier et al., 2009), oncogenic mutations (Aird et al., 2013), and metabolic stress in response to insults such as free radical release, hypoxia, and oxidative stress (Campisi and d'Adda di Fagagna, 2007). Cellular senescence thus provides a mechanism that prevents the undesirable proliferation of damaged cells, however, in contrast to their elimination through cell death mechanisms such as apoptosis, senescent cells remain viable, and continue to be metabolically active. Cell death and senescence can be triggered by the same stressors and we do not yet have a full understanding of what determines each cells fate (Herranz and Gil, 2018). Furthermore, whether particular injury stimuli can induce senescent cells with immediate features of chronic senescence remains unproven *in vivo*. The documented changes seen with acute and chronic senescence, including in the kidney, are summarized in **Figure 2**.

## Identification of Senescent Cells

The characterization of senescent cells *in vivo* remains challenging, in part because we have not yet identified a single marker that is specific to senescent cells. The signaling events that trigger a cell to become senescent vary depending on the senescence inducing stimuli with multiple pathways resulting in the induction of cyclin-dependent kinase inhibitors P16^INK4A^ and P21^CIP1^,leading to cell cycle arrest by enforcing the G1/S checkpoint. Increased senescence-associated β-galactosidase (SA-β-GAL), another important distinguishing characteristic of senescent cells, reflects the enhanced lysosomal content of senescent cells, though SA-β-GAL does not itself appear to be necessary for the senescence to occur (Hernandez-Segura et al., 2018). Importantly, the presence of any of these markers alone is insufficient to confirm senescence and false positives can occur. For example, P16^INK4A^ is not found in all senescent cells (Hernandez-Segura et al., 2017) and can be expressed in some non-senescent cells (Sharpless and Sherr, 2015). In addition, macrophages can express both P16^INK4A^ and SA-β-GAL (Hall et al., 2017). **Table 1** summarizes several of the positive and negative markers associated with senescence in the kidney. A recent publication from the International Cell Senescence Association has provided a consensus definition and three-step multi-marker workflow for detecting senescent cells (Gorgoulis et al., 2019). This approach takes account of the fact that while there are many potential markers of senescence, their specificity is context specific, and will be influenced by variety of factors including cell type and organizmal developmental stage.

**Table 1:** Tissue markers associated with senescence in the kidney.

Marker	Function	Expression levels in senescence/aging
**P16INK4A**	Cyclin-dependent kinase inhibitor 2A – blocks cell cycle at G1/S checkpoint	Increased after irradiation in the kidney (Freund et al., 2012), increased in tubular epithelial cells (Luo C. et al., 2018)
**P19ARF**	Cyclin-dependent kinase inhibitor 2A (alternate reading frame)– blocks cell cycle at G1/S checkpoint	Increased in the aging rodent kidney (Krishnamurthy et al., 2004)
**P21CIP1**	Cyclin-dependent kinase inhibitor 1A – blocks cell cycle at G1/S checkpoint	Increased. Seen in Human IgAN (Liu et al., 2012) and Rat Diabetic Nephropathy (Kitada et al., 2014)
**P27KIP1**	Cyclin-dependent kinase inhibitor 1B – blocks cell cycle at G1/S checkpoint	Higher levels of P27KIP1 linked to progression of diabetic nephropathy (Awazu et al., 2003)
**KI67**	Nuclear protein associated with cellular proliferation	Absent in senescent cells. Reduced with aging (Melk et al., 2009)
**SA-beta-gal**	Lysosomal beta-galactosidase	Increased with aging in the kidney (Baar et al., 2017)
**LMNB1**	Lamin-B1. Nuclear Lamina protein.	Decreased in human fibroblasts and in irradiated murine kidneys (Freund et al., 2012)
**Gamma-H2AX**	Component of DNA strand break repair machinery	Combination of high GammaH2AX+absent KI67 used as marker of DNA damage induced senescence in kidney transplants (Braun et al., 2012)
**SAHF**	Senescence associated heterochromatin foci	Reported with altered DNA packaging in senescent cells in the aging kidney (Lin et al., 2016)

The first step is to screen for senescence, by assessing SA-β-GAL activity. SA-β-GAL, however, cannot be used for paraffin-embedded tissue sections or in live cells, limiting its use. An alternative screening tool is to quantify lipofuscin, an emerging indicator of senescence which can be visualised in lysosomes *via* histochemical staining (Georgakopoulou et al., 2013; Evangelou et al., 2017).

The next step is to co-stain for additional markers of senescence. This includes P16^INK4A^ and P21^CIP1^, which are increased in senescence as well as the absence of proliferation markers and lamin-B1 (LMNB1), a major component of nuclear lamina (Shimi et al., 2011; Shah et al., 2013).

The third step is to identify senescence markers that would be expected to be altered in the specific biological context being studied. This includes evidence of DNA damage, which is associated with the cellular DNA damage response (DDR) (Munoz-Espin and Serrano, 2014) as well as FOXO, a transcription factor, and phosphoinositide 3-kinase (Pi3K), an intracellular signal transducer, both of which enable senescent cells to resist apoptosis (and hence, are potential targets for therapies to deplete senescent cells, discussed further below) (Hernandez-Segura et al., 2018). Finally, the secretome of senescent cells, collectively termed the senescence-associated secretory phenotype (SASP), can be quantified.

## The Senescence-Associated Secretory Phenotype

The senescence-associated secretory phenotype (SASP) includes a wide range of pro-inflammatory cytokines, chemokines, growth factors, and proteases which act in an autocrine and paracrine fashion on neighboring cells and are critical to understanding the impact senescent cells have on the function of tissues and organs (Coppe et al., 2008; Coppe et al., 2010; Laberge et al., 2012). Exploring these individually is out with the scope of this review, but excellent summaries are available in the current literature (Wang W. J. et al., 2017).

Established SASP components include but are not limited to IL-1β, IL-6, IL-8, GROα, TGFβ1, and WNT16B (Yang et al., 2006; Coppe et al., 2008; Kuilman et al., 2008; Binet et al., 2009; Ito et al., 2017). Studies examining the transcriptome of senescent cells (Hernandez-Segura et al., 2017) and proteomic analysis of the supernatant secreted from senescent cells (Basisty et al., 2020) have identified a much larger pool of potential SASP makers and demonstrated that there is significant heterogeneity in the SASP composition depending on the senescence inducer and cell type involved. Furthermore, single cell RNA-sequencing studies, where senescence has been induced in a single cell type *via* the same mechanism, has demonstrated significant variability exists in SASP expression in cells from different lineages (Wiley et al., 2017).

The SASP, to a large extent, is initiated through NF-κB, which itself is triggered by the DDR (Salminen et al., 2012). The Mammalian target of Rapamycin (mTOR) protein also has an important role regulating SASP production by promoting the activation of NF-kB *via* IL-1A translation (Laberge et al., 2015) and by inhibiting the breakdown of numerous SASP components by inhibiting the RNA-binding protein ZFP36L1 (Herranz et al., 2015).

The SASP mediates many of the beneficial and deleterious effects of senescent cells including inducing senescence in neighboring cells (Acosta et al., 2013). It contributes to a pro-inflammatory microenvironment that can activate immune cells and lead to the subsequent elimination of senescent cells (Munoz-Espin and Serrano, 2014). Why developmental and some forms of acute senescence are rapidly and completely cleared by senescent cell death and phagocytosis, while chronic senescence appears resistant to immune clearance remains an important unanswered question.

Emerging evidence indicates that extracellular vesicles (EVs) are also important mediators by which senescent cells impact on their microenviroment (Borghesan et al., 2019). EVs, including exosomes and microvesicles, are membrane bound structures that contain proteins, lipids, and RNA (Sagini et al., 2018). They have a crucial role in maintaining cellular homeostasis by excreting harmful DNA and inhibition of exosome secretion has been shown initiate DDR in both senescent and non-senescent cells (Takahashi et al., 2017). There is an increase in EVs secreted from senescent cells (Jeon et al., 2019) and they can induce paracrine senescence in neighboring cells (Borghesan et al., 2019).

## Metabolic Alterations in Senescence

Although “senescent” may be interpreted as “inert”, senescent cells retain an active but altered metabolism. Impaired mitochondrial oxidative phosphorylation is associated with both aging and senescence in nematode worms, experimental mice and man (Boffoli et al., 1994; Ishii et al., 1998; Trifunovic et al., 2004). It is recognized that signaling mechanisms exist by which mitochondrial cues can alter gene expression in the nucleus including Sirtuin-1 (Sirt1) and mammalian target of rapamycin (mTOR) expression, pathways implicated in cell survival and senescence induction (Kwon et al., 2019). One such signaling pathway is reactive oxygen species generation, itself a source of DNA, protein, and lipid damage and hence a potential senescence trigger (Shiloh, 2003).

Senescent cells also contain more glycogen granules—implicating a role for glycogenesis in the induction of senescence with inhibitors of glycogen synthase kinase 3 (GSK3) including TGFB1 release inducing senescence and glycogen accumulation (Seo et al., 2008; Byun et al., 2012). Protein synthesis and proteostasis are also key requirements for cellular health. Metabolic regulators GSK3, AMP-activated protein kinase (AMPK), and mTOR all modulate protein degradation and synthesis—and in senescence altered activation of these pathways results in abnormally enhanced protein synthesis (Mazucanti et al., 2015).

Finally, autophagy also plays a role in organelle quality control, and inhibition of GSK3 can impair autophagy and increase ROS generation(Byun et al., 2012). Of the three key signaling kinases, GSK3 and AMPK are positive regulators, with mTOR a negative regulator of the metabolic features of senescence.

## Physiological Roles of Senescence

### Developmental Senescence

Senescent cells have been identified in the developing embryos of multiple species including humans (Munoz-Espin et al., 2013), birds (Nacher et al., 2006), amphibians (Davaapil et al., 2017), and fish (Villiard et al., 2017) indicating the role of senescence in embryogenesis is highly conserved. In mice, senescent cells have been found in the apical ectodermal ridge of the developing limb, the mesonephros, the neural tube, and the endolymphatic sac of the inner ear (Munoz-Espin et al., 2013; Storer et al., 2013) with cells being identified on the basis of SA-β-GAL and P21^CIP1^ expression and SASP mediators. Notably, other markers of senescence such as P16^INK4A^ and markers of DNA damage were absent. Senescence is followed by macrophage infiltration, which clear the senescent cells as part of tightly controlled cellular process. It appears in every embryo, demonstrating that this is normal programmed behaviour and not a response to injury (Rhinn et al., 2019). The role of senescent cells appears to be in the fine tuning of organogenesis; P21^CIP1^ knockout mice with altered ability to induce senescence remain viable with a compensatory increase in apoptosis, while exhibiting detectable abnormalities in their kidneys, limbs, and vagina (Munoz-Espin et al., 2013; Storer et al., 2013).

A role for senescence in the timing of parturition has also been suggested (Cha and Aronoff, 2017; Menon et al., 2017). In pregnant mice with a conditional deletion of p53 in uterine tissues, half went into preterm labor (compared to none in controls) with increased markers of senescence including P21^CIP1^ in post implantation decidual cells, i.e., cells of the pregnant endometrium that form the maternal placenta (Hirota et al., 2010). Intriguingly, the same group demonstrated that this premature decidual senescence occurred as a result of increased mTOR complex 1 signaling and that the administration of the mTOR inhibitor Rapamycin reduced the incidence preterm birth (Hirota et al., 2011). In addition, it has been hypothesised that signaling from senescent cells in the foetal membrane to maternal uterine tissues can drive parturition (Menon et al., 2017).

### Wound Healing

Acute senescence is part of the regenerative response following injury. Murine studies have demonstrated that senescent endothelial cells and fibroblasts appear early in response to cutaneous injury and promote wound healing by secreting platelet-derived growth factor AA (PDGF-AA), a SASP mediator that induces myofibroblast differentiation. Senescent cell depletion delayed but did not prevent wound healing (Demaria et al., 2014). Other studies have demonstrated that senescent fibroblasts accumulate and act to limit fibrosis in the granulation tissue of healing skin wounds (Jun and Lau, 2010) and in the heart post myocardial infarction (Zhu et al., 2013). Senescent hepatic stellate cells also appear to limit fibrosis in the liver following injury (Krizhanovsky et al., 2008), however, when their subsequent Natural Killer cell-mediated depletion was impaired, their accumulation lead to increased fibrosis (Sagiv et al., 2016).

### Cancer Defense

Cell division is essential for growth and repair but tight physiological control is essential to prevent malignancy. Oncogene activation can induce senescence and act as a barrier to neoplastic transformation (Hinds and Pietruska, 2017). Genes related to cellular senescence are frequently mutated in cancer (Zhao et al., 2016). In studies of P21^CIP1^-deficient mice with alterations in their ability to undergo senescence, an increased susceptibility to multiple cancers was observed (Martin-Caballero et al., 2001). In humans, melanocytic nevi (moles), frequently harbor the oncogenic BRAF^V600E/K^ mutation, but only rarely progress into malignant melanoma as the BRAF^V600E/K^ mutation induces senescence with both P16^INK4A^ and SA-β-Gal evident in lesions (Michaloglou et al., 2005). Calcitriol, an active form of vitamin D, has anti-proliferative activity against a number of cancers including renal cancer. *In vitro* studies have attributed this effect to calcitriol inducing senescence with increased expression of P16^INK4A^ (Shen et al., 2017).

The paracrine effects of the SASP also play a key role. In addition to reinforcing growth arrest in neighboring cells, the SASP drives the recruitment and activation of immune cells, which then eliminate damaged cells. This process has been termed “senescent surveillance”. Murine pre-malignant senescent hepatocytes secrete chemokines and cytokines that drives the immune mediated clearance of pre-malignant cells. Impaired removal of senescent hepatocytes, promoted the development murine hepatocellular cancer (Kang et al., 2011).

However, the SASP can also have a pro-tumorigenic impact on pre-malignant cells and on the tumor microenvironment. Senescent human fibroblasts stimulate both pre-malignant and malignant epithelial cells to form tumors in mice (Krtolica et al., 2001). In addition, studies have demonstrated that the SASP from human senescent cells, particularly IL-6 and IL-8, induces an epithelial-to-mesenchyme transition (Coppe et al., 2008), which is strongly associated with tumor progression and subsequent tissue invasion (Ye and Weinberg, 2015). In a study using a mouse model where senescence can be induced in osteoblasts using tamoxifen, the authors found that this increased cancer spread from breast to bone by creating a favourable microenvironment for malignant cells. Notably, tumor burden was significantly reduced by the administration of IL-6 neutralising antibodies (Luo et al., 2016).

## Senescence in the Kidney

### Why is Senescence Relevant in Kidney Disease?

Older kidneys exhibit decreased function and increased susceptibility to acute kidney injury (AKI) (Ishani et al., 2009; Coca, 2010; Ferenbach et al., 2011; Clements et al., 2013; O'Sullivan et al., 2017). For many years, this was accepted as a fully recoverable entity; with serum parameters of kidney function returning to normal post-injury. We now understand from epidemiological data with both adequate statistical power and length of follow-up, that survivors of AKI have a significantly increased risk of developing chronic kidney disease (CKD). (Ishani et al., 2009; Coca et al., 2012; Ferenbach and Bonventre, 2015) CKD is generally progressive, even in circumstances where the original renal injury is no longer present or has been treated and is incurable; with a common progression pathway of multiple renal diseases being accumulating fibrosis.

An association between aging and increasing numbers of senescent cells has been noted in multiple organs including the kidney (Sis et al., 2007; Melk et al., 2009; Liu et al., 2012). A question which is the focus of intense ongoing research relates to whether these increased numbers of senescent cells merely represent a biomarker of aging or previous injury, or whether senescent cells actively promote maladaptive repair, organ fibrosis, and dysfunction, and hence represent a novel therapeutic target.

Senescent cells fulfil several criteria which make them novel candidate drivers of kidney disease. Namely, they are found in the context of tissue injury, are resistant to apoptosis and persist within the kidney secreting pro-inflammatory and pro-fibrotic signals which may contribute to progressive damage. (Melk et al., 2005; Sis et al., 2007; Braun et al., 2012; Liu et al., 2012) While key questions remain unanswered in the kidney, several recent papers using transgenic or pharmacological depletion of senescent cells in murine models support a causal role for senescence in progressive renal fibrosis (Baker et al., 2016; Baar et al., 2017; Kim et al., 2019).

**Figure 3:**
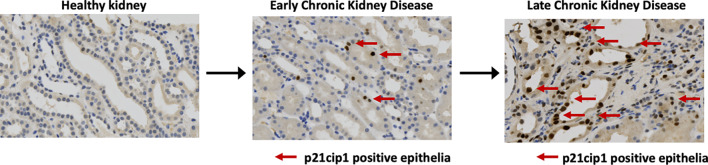
Senescent cells accumulate in progressive chronic kidney disease. Immunohistochemical staining for expression of the cyclin dependent kinase inhibitor P21CIP1 in native human kidney biopsies obtained at the Royal Infirmary of Edinburgh, Scotland illustrates increasing proportions of P21CIP1+ positive epithelial cells with advancing disease (red arrows).

Senescent cells accumulate in the kidney in three broad settings: with age, with any insult causing acute kidney injury, including post-transplantation, and in chronic kidney disease. In each case, higher levels of senescent cells associate with worsened kidney function and outcome (**Figure 3**).

The tubular epithelium is implicated as the primary location for renal senescence. This is demonstrated by both human and murine data: In Baker et al's seminal study of ablation of senescent cells in INK-ATTAC transgenic mice, they demonstrated senescence occurring in proximal tubules with increasing age (Baker et al., 2016). In a series of human renal transplant biopsies, all biopsy specimens showed P16^INK4A^ staining in the nuclei of distal tubules and collecting duct but staining was also present in podocytes, parietal epithelium of glomeruli, vascular smooth muscle cells, and interstitial cells (Melk et al., 2005). Furthermore, a series of patients with glomerular disease showed P16^INK4A^ staining in a subset of glomerular, tubular, and interstitial cell nuclei; however, senescent tubular epithelial cells were the key difference between disease and control kidneys, present in 80% of cases compared with 21% in healthy living donor control kidneys (Sis et al., 2007). Additionally, a biopsy series of patients with IgA nephropathy demonstrated increased P21^CIP1^ and P16^INK4A^ protein expression which was confined to tubules (Liu et al., 2012).

**Figure 4:**
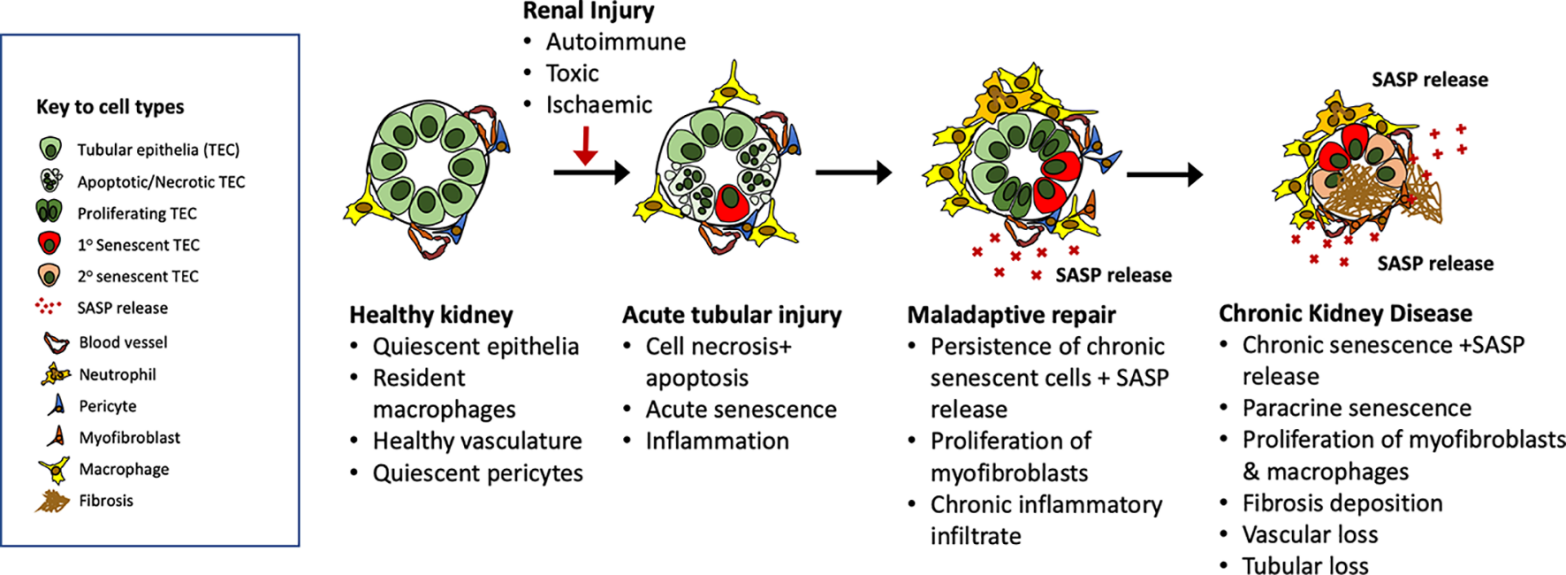
Putative roles for senescent cells in the evolution of maladaptive repair after acute kidney injury. Our understanding of the role of senescence in the evolution of maladaptive repair after injury remains incomplete. Evidence from clinical studies identifies increasing numbers of senescent cells in the aged and chronically damaged kidney, and experimental models show reduced renal fibrosis after senolytic treatments. In this diagram, we outline potential mechanisms by which senescent cells generated in response to aging or injury may promote ongoing fibrosis *via* SASP release leading to immune activation, fibroblast proliferation, and further induction of paracrine senescence.

As discussed earlier in this review and elsewhere, there is an increasing appreciation of the prevalence of maladaptive repair in the aftermath of kidney aging and injury (Ferenbach and Bonventre, 2015) leading to fibrosis within the kidney. Based on the available evidence in the kidney and beyond we propose that in the aftermath of renal injury, chronically senescent renal epithelial cells mediate progressive fibrosis, vascular rarefaction and organ dysfunction *via* SASP release (**Figure 4**).

### Pathological Roles of Senescence Beyond the Kidney

In contrast to the tightly controlled and beneficial roles outlined earlier in this review, the accumulation of senescent cells in aging is now known to be detrimental, with their clearance extending median healthspan in mice by an average of 40% (equating to > 30 years in humans) (Xu et al., 2018).

From an evolutionary perspective healthy aging does not undergo selection pressure, i.e., traits of senescence conferring improved health to the point of reproduction undergo positive selection (cancer defense, embryogenesis, parturition), with no selection for potential detrimental late life effects. Thus, while induction of senescent cells may improve early wound repair, conversely their SASP release may contribute to tissue dysfunction in aging and pathological conditions; post-injury and in disease.

There are now striking examples across tissues and diseases of senescent cell clearance being beneficial. Much of our early understanding of senescent cell removal derives from transgenic approaches which have used components of the cyclin-dependent kinase 2A gene (P16^INK4A^) to drive concomitant expression of a “suicide gene” activated upon administration of a drug or dietary compound, thus conferring selective vulnerability upon P16^INK4A^ expressing cells to pharmacological clearance (Baker et al., 2011; Baker et al., 2016; Chang et al., 2016). Although informative, the results of studies based exclusively upon such transgenes may not fully recapitulate the spectra of senescent cells seen *in vivo*, unless all such cells express P16^INK4A^, which seems unlikely.

### Senescence and Fibrosis

As described in detail above, the SASP comprises pro-fibrotic and pro-inflammatory molecules. Thus, it seems plausible, and indeed likely, that part of the deleterious effect of SC accumulation is mediated *via* tissue fibrosis. The means by which SCs exert a pro-fibrotic effect within an organ are yet to be fully defined. However, there are clear examples of this effect already in published literature.

Cellular senescence has been shown to be important in the pathogenesis of idiopathic pulmonary fibrosis; a progressive fibrotic lung disease with devastating consequences on both mortality and morbidity. (Faner et al., 2012; Hashimoto et al., 2016) Senescent cell depletion (either by transgenic means, or by pharmacological depletion by dasatinib and quercetin) resulted in healthier, less fibrotic lungs in mice.

An association between cellular senescence and resultant fibrosis has also been shown in the liver, where transgenic induction of senescence significantly exaggerates fibrosis in the context of acute injury. (Ferreira-Gonzalez et al., 2018) Similarly, in the kidney, transgenic depletion of SCs in the INK-ATTAC model resulted in significantly less glomerulosclerosis (Baker et al., 2016).

## Modulation of Senescence in Health and Disease

### Animal Models of Senescent Cell Deficiency or Depletion in the Kidney

Our understanding of senescent cell accumulation and clearance in renal disease remains limited. To date, there are three key experimental strategies which have been used to test the manipulation or removal of senescent cells. These are: transgenic mice with alterations in global senescence induction pathways, transgenic approaches to deplete senescent cells, and pharmacological targeting of senescent cells and/or their secretome (Megyesi et al., 2001; Wolstein et al., 2010; Baker et al., 2011; Munoz-Espin et al., 2013; Baker et al., 2016; Chang et al., 2016; Baar et al., 2017; Xu et al., 2018; Kim et al., 2019).

### Studies in Mice With Transgenically Modified Senescence Induction Pathways

Over the course of the last 20 years, several studies of kidney injury and disease have been published using transgenic mice with alterations in senescence induction pathways (summarized in **Table 2**); predominantly global P16^INK4A^ and P21^CIP1^ knockout models. These models have used several models of experimental renal injury; including ischaemia reperfusion injury (IRI), unilateral ureteric obstruction (UUO), diabetic nephropathy (DN), and post transplantation with mixed results; with an approximately even split between benefit and harm (Al-Douahji et al., 1999; Megyesi et al., 2001; Wolf et al., 2005; Wolstein et al., 2010; Braun et al., 2012; Megyesi et al., 2015).

**Table 2:** Transgenic murine models of altered senescence induction and their outcomes in the kidney.

Reference	Renal model	Modulation of senescence	Renal Disease outcome	Effect of any Intervention
Baker et al. (2016)	Natural aging In INK-ATTAC mice	INK-ATTAC +AP20187 or vehicle administration to deplete P16INK4A+ cells	↑Glomerulosclerosis ↑ ß-gal positivity	↓Glomerulosclerosis ↓ß-gal positivity
Baar et al. (2017)	Natural aging P16-3MR mice and fast aging Xpd TTD/TTD mice	FOXO4-DRI agent causes p53 nuclear exclusion. Ganciclovir Rx to P16-3MR mice causes P16INK4A+ restricted cell death	↑Serum Urea ↓Lmnb1 expression ↑SASP expression (both Xpd^TTD/TTD^ and aged P16-3MR)	FOXO4-DRI or GCV to P16-3MR admin: ↓Serum Urea ↑Lmnb1 expression ↓SASP expression (both Xpd^TTD/TTD^ and aged P16-3MR)
Munoz-Espin et al. (2013)	Nephrogenesis	WT vs P21CIP1 KO mice with deficient growth arrest in nephrogenesis	↓ ß-gal positivity in P21CIP1 KO mice utero. ↑Ki67 expression but ↑Apoptosis maintains development	Use of PI3K inhibitor augments developmental senescence in WT mice
Wolstein et al. (2010)	UUO	WT vs P16INK4A KO mice with impaired cell cycle arrest	UUO induces ß-gal positivity, apoptosis and collagen deposition in WT mice	↓ ß-gal positivity ↓Apoptosis ↑Collagen, ↑proliferation after UUO in P16INK4A KO
Megyesi et al. (2001)	Renal IRI	WT vs P21CIP1 KO mice with impaired cell cycle arrest	WT mice show tubular injury and raised blood urea levels after IRI	↑proliferation ↓Renal function ↑Mortality in P21CIP1 KO
Megyesi et al. (2015)	Renal UUO and IRI	WT vs P21CIP1 KO vs P21CIP1 KO+KAP2-driven P21CIP1	WT mice show renal fibrosis after both UUO and IRI	UUO: ↓fibrosis in P21CIP1 KO vs WT, ↑fibrosis in P21CIP1 KO kidneys with KAP2 promotor driven P21CIP1 vs P21CIP1 KO. IRI: ↑TGFß1 in P21CIP1 KO kidneys with KAP2 promotor driven P21CIP1 vs P21CIP1 KO.
Lee et al. (2012)	Renal IRI	WT vs P16INK4A/p19ARF Double KO mice with impaired cell cycle arrest	WT mice show marked P16INK4A and p19ARF induction 28d after IRI, with apotosis and reduced tubular density	P16INK4A and p19ARF deficient mice show improved epithelial and microvascular repair, with increased myeloid cell recruitment
Al-Douahji et al. (1999) Wolf et al. (2005)	Diabetic Nephropathy	WT vs P21CIP1 KO WT vs p27KIP1 KO	WT mice develop albuminuria and glomerular hypertrophy	Both p27KIP1 KO and P21CIP1KO mice were protected from proteinuria and glomerular expansion
Braun et al. (2012)	Renal Transplant	P16INK4A KO mice with impaired cell cycle arrest	WT mice develop interstitial fibrosis and tubular atrophy	P16INK4A KO mice develop less atrophy and fibrosis after Tx

In each case ↑ ↓ markers were used to indicate that the p value was less than 0.05.

P16^INK4A^ knockout mice exposed to experimental renal injury show increased epithelial proliferation and functional recovery after IRI but worsened fibrosis in UUO models. This implies that senescent cells can behave differently in response to altered injury cues with their generation both time and population determining their subsequent influence on tissue repair and fibrosis (Wolstein et al., 2010; Lee et al., 2012).

### Transgenic Approaches to Deplete Senescent Cells

An alternative experimental approach to inhibiting the induction of senescence is to selectively deplete cells only after they have become senescent. As senescent cells are programmed to survive stress signals that would lead to cell death in normal proliferating cells; published transgenic approaches thus far use a senescence promoter; typically, the promoter for the P16^INK4a^ gene, to drive the expression of a concomitant suicide gene which can subsequently be activated by administration of a drug or dietary compound.

In the 2016 study by Baker et al. (2016), senescent cell depletion was achieved by using P16^INK4A^ promoter-driven expression of FK506-binding-protein-Caspase8; a transmembrane receptor coupled to an intracellular caspase pathway which was activated upon administration of AP20817 (AP). (Baker et al., 2016) Successful elimination of senescent cells in the kidney was confirmed by SA-β-Gal staining with a 75% reduction in the numbers of senescent cells observed by transmission electron microscopy.

The kidneys of mice who underwent senescent cell depletion showed significantly less glomerulosclerosis, and lower blood urea levels. Of note, despite improved glomerular appearance; senescent cells were located primarily in the tubular epithelium and not in the glomeruli. Interestingly, AP-treated mice showed decreased levels of angiotensin receptor 1a (atr1a), both at transcript and protein level. In humans, angiotensin receptor blockers attenuate age and disease-related glomerulosclerosis under normotensive conditions and are used to control proteinuria; leading to the theory that over-activation of the local renin-angiotensin-aldosterone system (RAAS) drives glomerulosclerosis. (Ferder et al., 2003; Paul et al., 2006) The reduction of receptor expression in mice with lower numbers of senescent tubular epithelial cells implicates a senescence driven activation of the renal RAAS as potentially contributory to the blood pressure changes seen with aging and in human CKD.

A similar approach was subsequently used by Baar et al. using the P16:3MR mouse model, (Baar et al., 2017) where the P16^INK4a^ promoter drives the expression of a viral thymidine kinase, conferring selective sensitivity to the effects of the antiviral drug Ganciclovir. Mice who underwent senescent cell depletion by means of Ganciclovir treatment had lower levels of plasma serum urea and creatinine.

Though powerful in terms of informing future direction of study, transgenic depletion and the global knockout (KO) models described above must be interpreted in the knowledge that a generic P16^INK4a^ (or P21^CIP1^) promotor driven genetic KO is neither specific to senescent cells, nor captures all potential senescent cells, i.e., some non-senescent cells express P16^INK4a^ and some senescent cells do not express P16^INK4A^.

Both these transgenic models (INK-ATTAC and P16:3MR) have been used to study disease in other organs and have demonstrated benefit post depletion in several models of disease including bone marrow irradiation, tau-mediated neurodegeneration, and bleomycin induced pulmonary fibrosis (Chang et al., 2016; Schafer et al., 2017; Bussian et al., 2018).

### Pharmacological Depletion of Senescent Cells

Studies of murine models with defects in senescence induction and transgenic methods of senescent cell depletion have made major contributions to our understanding of the role played by senescence in aging and disease. In order to translate studies from the lab to the clinic there is a requirement for pharmacological agents capable of manipulating the survival and/or function of senescent cells.

**Figure 5:**
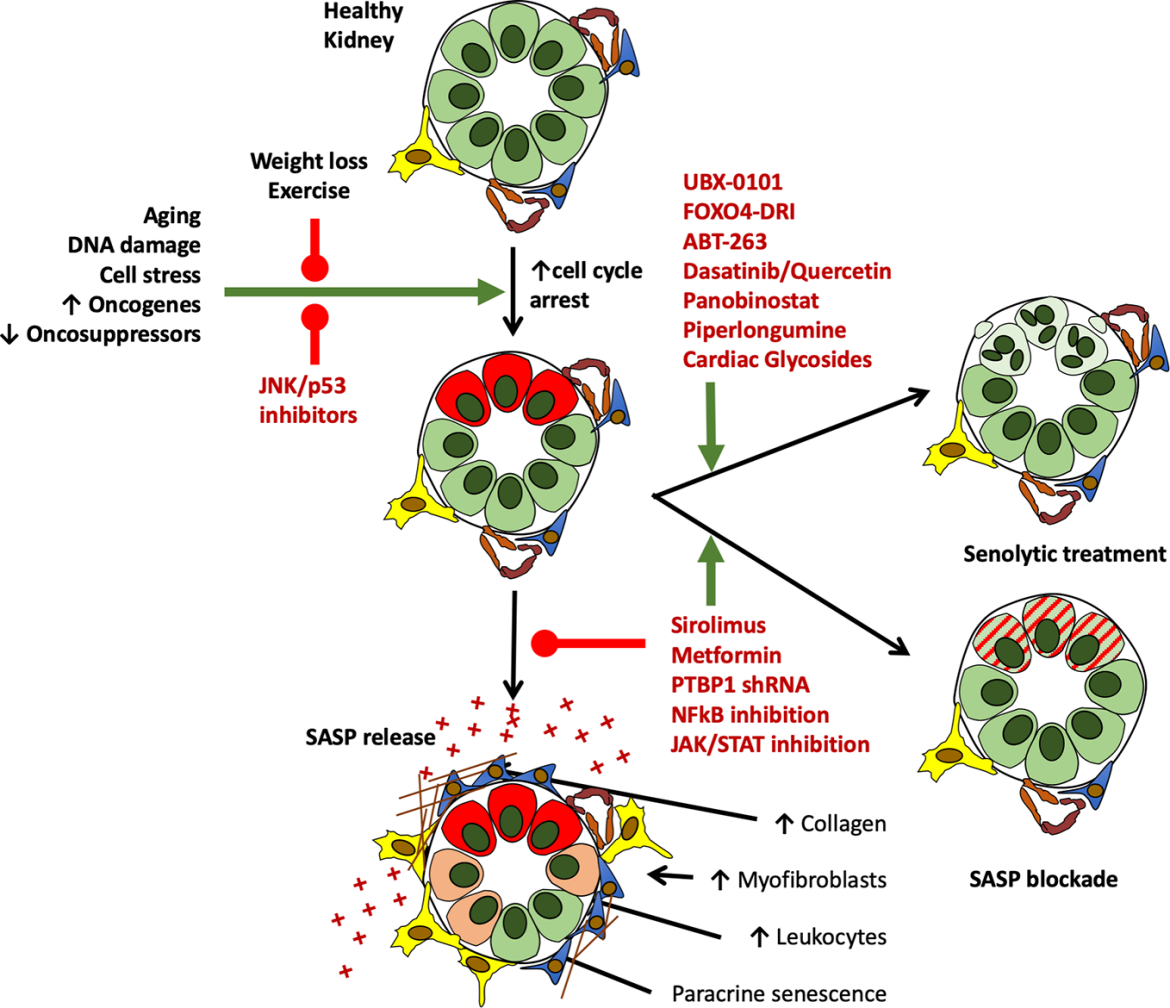
Current and potential future interventions to target growth arrested cells in the kidney *in vivo*. While at present no drugs are FDA approved for the treatment of senescence, study of the mechanisms leading to senescence induction, and its downstream deleterious consequences of SASP release have led to the identification of several routes to target senescent cells and their effects. Drugs targeting senescent cell generation, depletion, and their functional alteration are shown in red below. Of note, several agents on this list are clinically licenced for other indications, offering potential routes toward clinical translation.

Chronically senescent cells are typically able to resist apoptosis. They have commonly survived a significant cell stress and entered into permanent growth arrest and have thus often up-regulated anti-apoptotic pathways (Childs et al., 2014; Zhu et al., 2015; Soto-Gamez et al., 2019). By blocking or interfering with these pathways it has proved possible to induce senescent cell apoptosis (Baar et al., 2017). Though still in its relative infancy, this is an approach with both marked experimental value and enormous translational potential at both an organ and whole animal level. Experimental pharmacological depletion of senescent cells has been shown to improve outcomes in organs other than the kidney across several models of disease and has been shown to not only improve median lifespan, but also reduced late life morbidity (Xu et al., 2018; Hickson et al., 2019). Several agents have been shown to have efficacy in modulating senescent cell number and/or function in an experimental setting. Published literature thus far suggests that different senolytic agents are efficacious against different cell types (Xu et al., 2018). Common agents which have been used in a variety of *in vivo* and *in vitro* models include FOXO4-DRI, ABT-263 (Navitoclax), and the combination of dasatinib and quercetin (D+Q) (Chang et al., 2016; Baar et al., 2017; Schafer et al., 2017; Bussian et al., 2018; Xu et al., 2018). These and other agents under investigation are discussed below (**Figure 5**).

#### FOXO4-DRI

While at present, little data exist which report specifically on renal outcomes after pharmacological depletion, in 2017, Baar et al. identified the FOXO4 protein as an important regulator of p53 activity in senescent cells. The p53 pathway is an important controller of apoptosis and senescence and as such is a potential target for senolytic treatment. FOXO4 was hypothesised to bind with p53 and thus prevent p53 mediated apoptosis. By designing an interfering peptide “FOXO4-DRI”, which competes with endogenous FOXO4 for p53 binding, they demonstrated that selective apoptosis could be achieved in both aging and chemotherapy-induced senescent cells. Fast-aging mice (a murine model of the human autosomal dominant disease trichothiodystrophy; in which mice are deficient in DNA repair) and mice with Doxorubicin-induced senescence post-chemotherapy that were treated with FOXO4-DRI were less frail, more responsive and had significantly improved “healthspan” compared to mice who had not undergone senescent cell clearance (Baar et al., 2017).

In keeping with expected age-related decline in kidney function; fast-aging mice had higher urea levels when compared to wild-type counterparts and when assessed specifically for senescence, kidneys from fast-aging mice were demonstrated to have significantly higher levels of SA-β-Gal and loss of LMNB1. (Freund et al., 2012) Again, senescent cells were primarily located in the tubular epithelium. (Baker et al., 2016) Staining for IL-6, a known SASP component, was also increased.

Treatment with FOXO4-DRI significantly reduced the percentage of tubular cells lacking in LMNB1 and resulted in lower blood urea levels and lower IL-6 expression. As the mechanism of this approach is to induce cell death, it is reassuring to note that there was no significant change in overall kidney weight in the treated group; thus, averting concern of widespread tissue death and loss of tissue architecture. Indeed, this maintenance of weight (in the presence of confirmed cell loss) suggests regeneration. This is a potentially similar phenomenon to that seen in bone marrow, where depletion of senescent cells improves marrow clonogenicity (Chang et al., 2016).

#### ABT-263 (Navitoclax)

As aforementioned, senescent cells are able to evade apoptosis (Zhu et al., 2015). One route to achieving this is through the up-regulation of pro-survival proteins; Bcl-2, Bcl-w, and Bcl-xL. These proteins exert their anti-apoptotic effects by direct binding actions that regulate mitochondrial outer membrane permeabilisation (MOMP). ABT-263 (and related compounds ABT-737 and ABT-199) is a Bcl-2/w/xL inhibitor (Tse et al., 2008) which attaches to the BH3-binding groove on these molecules, thus preventing the binding of pro-apoptotic proteins. These proteins therefore remain in an active (unbound) state and are capable of mitochondrial membrane permeablisation and apoptosis (Kale et al., 2018).

Sub-lethal total body irradiation induces bone marrow aging in mice and has been shown to induce haematopoietic stem cell (HSC) senescence. Treatment with ABT-263 reduces haematopoietic senescent stem cell number. Although Chang et al. showed that ABT-263 was efficacious against senescent renal epithelial cells, the impact of ABT-263 treatment on renal function and fibrosis remains unknown (Chang et al., 2016).

HSCs subjected to senescence induction by irradiation are phenotypically similar those found in aged mice. Treatment of irradiated mice or naturally aged mice was shown to rejuvenate irradiated marrow such that when transplanted into a healthy recipient (i.e., non-irradiated) both clonogenicity and long-term engraftment were improved, potentially *via* an improvement in the stem cell microenvironment (Chang et al., 2016).

Similarly, in both murine models of neurodegenerative disease and atherosclerosis, treatment with ABT-263 resulted in an attenuated disease phenotype with significantly less tau phosphorylation and reduced atherosclerotic burden. (Childs et al., 2016; Bussian et al., 2018) ABT-263 can lead to a thrombocytopaenia (Kaefer et al., 2014) which has limited its prolonged use as an antineoplastic agent. Whether a short period of treatment would be sufficient to deplete senescent cells and impact meaningfully on subsequent clinical outcomes in man remains to be determined.

Additionally, within this family (i.e., the BH3 mimetics), there exist differences in the efficacy of senolysis between specific drugs and this remains of therapeutic interest. Both senescent cells, malignant cells, undifferentiated stem cells, and megkaryocytes show selectivity in response to different members of this class of drug, related to varying expression of different members of the Bcl-2 family. For example, venetoclax (ABT-199); a selective Bcl-2 inibitor which is used in the treatment of multiple myeloma has been shown to be effective in the setting of high Bcl-2 expression with concomittant low Bcl-XL expression and cells which co-express Bcl-2 and Bcl-XL to similar levels are resistant to venetoclax and require additional treatment with a Bcl-XL inhibitor (Punnoose et al., 2016).

Inhibition of Bcl-XL and Bcl-W *in vitro* and *in vivo* has been shown to induce apoptosis of senescent cells, whereas Bcl-2 inhibition alone had no significant senolytic effect. This suggests that ABT-263 or ABT-737 may be better therapeutic choices in terms of senolytics. (Yosef et al., 2016) It is worth noting, as mentioned above, Bcl-XL is a key pro-survival protein involved in the maintenance of platelets; thus explaining the resultant thromobocytopenia observed with ABT-263 and not seen with ABT-199 (Kile, 2014).

#### Dasatinib and Quercetin

Transplantation of senescent cells into healthy young mice has been shown to induce physical dysfunction, and earlier death. Depletion of senescent cells in this context with combination of dasatinib and quercetin (D+Q) attenuated the deterioration in walking speed, hanging endurance, and grip strength with similar beneficial effects on frailty in naturally aged mice (Xu et al., 2018).

With respect to mechanism of action; D+Q are proposed to have multiple intracellular targets. (Paez-Ribes et al., 2019) Dasatinib is a tyrosine kinase inhibitor which inhibits Ephrin B (EFNB)-dependent suppression of apoptosis, and has been shown to preferentially decrease viability and increase cell death in senescent compared to non-senescent human pre-adipocytes. (Zhu et al., 2015) Quercetin is a naturally occurring flavonoid which inhibits phosphoinositide 3- kinase (PI3K) and has been shown to induce cell death in senescent human umbilical vein endothelial cells (HUVECs) (Zhu et al., 2015).

Old mice (24-27 months) treated intermittently with D+Q had a 36% increase in median lifespan, with no reduction in physical function in that time (Xu et al., 2018). A single administration of D+Q in aged mice was sufficient to improve cardiovascular function and also reduced the expression of P16^INK4A^ and prevalence of SA-β-Gal positive cells after localised limb irradiation (Zhu et al., 2015).

Senescent cell depletion with D+Q has also been shown to be of benefit in a murine model of bleomycin-induced pulmonary fibrosis, with treated mice demonstrating an improvement across several markers of lung, and whole animal health; including reduced pulmonary resistance (as measured by enhanced pause, an indirect measurement of lung compliance in mice), preserved body weight, and improved exercise capacity. (Schafer et al., 2017) Despite improvements in lung function following senescent cell clearance, quantified reductions in lung fibrosis histopathology however did not reach statistical significance.

Mice exposed to six months of high-fat diet (HFD) have been shown to develop increased renal cellular senescence and increased fibrosis. Prolonged (ten week) treatment with oral Quercetin reduced several markers of senescence including SA-β-Gal staining, whole kidney expression of p16, p19, and p53, and expression of classic SASP factors CCL2 and Il-1α. However, there was no significant difference in p21 (CKD1a) expression between groups; i.e., no increased p21 expression in the context of HFD and no significant change post-treatment with quercetin. Mice treated with quercetin were found to have significantly less tubular epithelial cell apoptosis and had similar levels of creatinine and microalbuminuria to wild type littermate controls (vs HFD mice treated with vehicle). Quercetin was also shown to reduce the renal cortical hypoxia (as measured by magnetic resonance imaging, MRI) found in HFD mice. With respect to fibrosis; mice treated with quercetin had less interstitial fibrosis as quantified by Masson's trichrome staining, but no significant difference in collagen-1 expression as measured by immunofluorescence. (Kim et al., 2019) In another study using D+Q in obese mice, senolytic therapy lead to an improvement in microalbuminuria and in increased expression of Wilms tumor protein (Wt‐1), which is a measure of podocyte integrity and function (Palmer et al., 2019).

To date, there have been three studies of senolytics published in humans, with several more on-going. Thus far, two trials have shown D+Q to be safe and well-tolerated in the setting of idiopathic pulmonary fibrosis and in diabetes (Hickson et al., 2019; Justice et al., 2019). Furthermore, D+Q were effective in reducing senescent cell number in adipose tissue thus suggesting that the senolytic effects observed in mice are reproducible in humans (Hickson et al., 2019). An additional single arm study, used Dasatinib alone in patients with systemic sclerosis associated interstitial lung disease (Martyanov et al., 2017). A *post hoc* re-examination of the results from 12 patients found a decrease in the skin expression of SASP markers with treatment that correlated with clinical improvement (Martyanov et al., 2019).

#### Piperlongumine and Panobinostat

Piperlongumine is a biologically active component of the *Piper Longum* pepper plant with reported senolytic properties (Bezerra et al., 2013). It exhibits wide-ranging biological actions including anti-cancer, anti-inflammatory, and senolytic properties, even at very small dose, making this a potentially important compound in terms of limiting toxicity to healthy cells (Bezerra et al., 2013). Piperlongumine regulates cell signaling *via* receptor tyrosine kinase (Raf-1) and extracellular signal-regulated kinases ERK1/2. It has been shown to have moderate senolytic activity against senescent human fibroblasts *in vitro* (Wang et al., 2016).

Panobinostat is a histone deacetylase inhibitor (HDACi) has been shown to have senolytic activity in the setting of Cisplatin-induced senescence in non-small cell lung cancer (NSLC), and in head and neck squamous cell carcinoma (HNSCC) cell lines (Samaraweera et al., 2017). HDACis have been shown to mediate reduction of Bcl-xl activity and thus in part, this may explain the senolytic effect of panobinostat (Duan et al., 2005; Samaraweera et al., 2017).

#### Cardiac Glycosides

Most recently, the cardiac glycosides digoxin, proscillaridin A, and ouabain have all been shown to be exert senolytic activity when tested against primary lung adenocarcinoma and fibroblast cell lines induced to senescence by Bleomycin *in vitro* (Triana-Martinez et al., 2019). Digoxin proved to be an effective senolytic in the context of *ex vivo* senescent chondrocytes from osteoarthritic donors. One proposed mechanism of action is inhibition of the Na+/K-ATPase pump and subsequent imbalance in cellular sodium and potassium, with senescent cells being primed by an already slight increase in depolarisation of the plasma membrane; thus, lowering the threshold for cell death when exposed to digoxin.

Oubain has also been shown to be a broad spectrum senolytic, effective against murine hepatic senescent cells and in adamantinomatous craniopharyngioma; a pituitary paediatric tumor in which clusters of β-catenin + pre-neoplastic senescence cells positively influence tumor progression in a paracrine manner (Gonzalez-Meljem et al., 2017; Guerrero et al., 2019).

The senolytic effect of cardiac glycosides remains to be fully determined. However, transcriptomic analysis performed by Guerrero et al. revealed that ouabain and digoxin activate the expression of genes of the pro-apoptotic BCL2 family. Among those, NOXA is induced by the JNK, GSK-3b, and P38 pathways, and partially mediates the senolytic effects of CGs (Guerrero et al., 2019).

#### Inhibition of G2/M Cell Cycle Arrest

As described, most senescence-inducing stimuli result in the induction of the cyclin- dependent kinase inhibitors p16ink4a and/or p21cip1 which enhance check- point activity, inducing cell-cycle arrest at the G1/S cell-cycle checkpoint with induction of the senescent phenotype. However, important work by the Bonventre group (Yang et al., 2010) has identified that accumulation of cells arrested at the later G2/M check point in the aftermath of kidney injury results not only in senescence, but triggers a pro-fibrotic secretory phenotype. This arrest is mediated *via* inhibition of cdc25c/cdc2 by p53 or by activation of the Chk1 and Chk2 proteins. Mimicking results seen in transgenic mice with reduced P16^INK4A^ and P21^CIP1^ leading to reduced G1/S cell cycle arrest—they employed JNK, histone deacetylase, and p53 inhibitors to reduce G2/M growth arrest and reduce renal fibrosis after ischaemic injury (Yang et al., 2010; Jenkins et al., 2014; Luo Q. et al., 2018).

Replicative senescence, and senescence induced by the DNA-damage response pathway (DDR) can also be induced at the G2/M checkpoint. (Mao et al., 2012; Jullien et al., 2013) Delineating the differences and commonality between G1/S and G2/M arrested cells, and the interplay between both checkpoints, will be of interest in the future and has potential implications for pharmacological inhibition of senescence; for example inhibition of movement of the cell through G1/S may reduce the number of arrested cells as G2/M.

### Pharmacological Targeting of the SASP—“Senostatics”

The SASP is a pro-inflammatory response that activates and reinforces senescence, modulates fibrosis and promotes regeneration (Acosta et al., 2008; Coppe et al., 2008). As such, it is an important facet of senescent cells in terms of their ability to influence whole organ or whole animal effects and it is therefore a potential therapeutic target for drugs (termed “senostatics”) which rather than killing senescent cells, instead impede their ability to release deleterious SASP molecules.

However, there are several important caveats to this approach. Firstly, the SASP is context dependent and dynamic (Coppe et al., 2008) and an approach to block or manipulate individual components of the SASP would require consideration of this. Secondly, the SASP comprises a vast array of secreted molecules and at present it is not clear which are pathogenic or how they induce damage. Finally, targeting the SASP mandates that treatment would likely be continuous as SASP production would continue unless senescent cells themselves were removed. In order to speed clinical translation of potential drugs, potential repurposing of existing clinically licenced agents merits attention.

#### NF-kB Inhibition

The NF-kB pathway is central to inflammation and has been proposed as a master regulator of the SASP (Chien et al., 2011). A cross-species study, which used motif mapping in promoters of genes upregulated with aging, suggested that NF-κB is the transcription factor most associated with aging (Adler et al., 2007).

While the full effect of NF-kB inhibition remains untested, it seems likely that with such a central role in cell signaling and inflammation, inhibition is likely to be context and cell dependent but has been proposed as a potential senolytic target (Salminen et al., 2012).

Drugs which have been considered for this purpose include antioxidants and inhibitors of the NF-kB pathway (Nelson et al., 2012; Tilstra et al., 2012). Genetic reduction of NF-kB in a fast aging mouse was shown to delay the onset of several age-related pathologies.

Likewise, pharmacological inhibition of NF-kB signaling can be achieved by using an inhibitor of the NF-kB activating kinase IKK. As is the case with genetic depletion, treated mice showed a delay in the onset of the majority of aging symptoms (Tilstra et al., 2012).

Furthermore, calorie restriction (CR) is widely recognized to extend longevity and in mice reduces senescent cell burden (Colman et al., 2008; Anderson et al., 2009; List et al., 2016). The beneficial effects of CR are not fully known but are in part attributed to suppression of NF-kB. CR inhibits NF-kB signaling at the level of the IKK complex. Short term CR resulted in a decrease in NF-kB activity in the kidneys of aged rats (Jung et al., 2009). Thus, CR; a known mediator of improved healthspan and a known senolytic approach appears to function at least in part through NF-kB inhibition.

#### JAK/STAT Inhibition

The SASP is rich in pro-inflammatory cytokines and chemokines. The JAK/STAT pathway plays an important role in regulating cytokine production (Yu et al., 2009; Meyer and Levine, 2014; Xu et al., 2015). Consistent with this, old rats have increased numbers of senescent pre-adipocytes and have higher levels of activated JAK1 and JAK2 (Xu et al., 2015). Treatment of senescent pre-adipocytes and HUVECs *in vitro* with JAK1 or JAK2 inhibitors significantly suppressed mRNA levels of key SASP components; including IL-6, IL-8, MMP12, and MMP3 (with little effect on control non-senescent cells). Similarly, *in vivo* old mice treated with Ruxolitinib (selective JAK1/2 inhibitor) resulted in reduced levels of cytokine expression otherwise normally associated with age; including IL-6 and CXCL-1 (Xu et al., 2015).

#### PTRP1 Inhibition

A recent study undertook an RNAi-based approach to identify transcriptional factors mediating the SASP profile of senescent ells. Using this approach, PTRP1 was identified as a factor mediating the inflammatory effects of the SASP with blockade inhibiting the pro-inflammatory and tumorigenic properties of senescent cells in the liver (Georgilis et al., 2018).

#### mTOR Inhibition

The mammalian target of rapamycin (mTOR), a serine/threonine kinase that plays central roles in various biological processes and has been shown to be important in some forms of senescence (Antonioli et al., 2019). Indeed, sirolimus (also known as rapamycin); a drug well known to nephrologists, has been shown experimentally to inhibit the senescence phenotype (Gu et al., 2016; Lesniewski et al., 2017; Wang R. et al., 2017). Given the importance of senescent cells in wound healing (Demaria et al., 2014), it is interesting to consider whether a previously unrecognized effect on senescence inhibition may have contributed to the impaired wound healing associated with clinical sirolimus use, though this remains to be proven.

#### Metformin

Similarly, metformin; another well-known drug with over 60 years' worth of clinical use is now being considered as an encouraging senostatic candidate. Metformin targets multiple intracellular signaling pathways closely associated with the development of aging, such as inflammation, cellular survival, stress defence, autophagy, and protein synthesis. It stimulates adenosine monophosphate‐activated protein kinase (AMPK); a pathway known to limit cellular stress and protect from oxidative stress (Fang et al., 2018; Piskovatska et al., 2019) and inhibits NF-kB pathway (Kanigur Sultuybek et al., 2019).

#### Blockade of Individual SASP Components

An alternative option is to consider manipulating individual SASP components. TGFβ is a well-recognized SASP component and a known driver of fibrosis (Acosta et al., 2013; Meng et al., 2016). Clinically relevant TGFβ blockers are available (Neuzillet et al., 2015) and inhibition of TGFβ signaling with Galunisertib has been shown to be effective in animal models of biliary disease and acute liver injury by blocking paracrine senescence leading to subsequent hepatocyte proliferation and improved liver function (Bird et al., 2018; Ferreira-Gonzalez et al., 2018). Connective tissue growth factor (CTGF, also known as CCN2) is another pro-fibrotic SASP mediator (Valentijn et al., 2018). In a phase 2 trial in patients with idiopathic pulmonary fibrosis, pamrevlumab, a fully recombinant human monoclonal antibody against CTGF, attenuated the decline in lung function and reduced the proportion of patients with disease progression compared to placebo (Richeldi et al., 2020).

### Future Directions

Fundamental questions remain surrounding the pathways regulating senescence, their roles in tissue aging and dysfunction, and their potential therapeutic use:

#### How Does the Site and Timing of Senescence Influence Outcomes?

Our understanding of senescent cells *in vivo* remains incomplete. Global knockout models and generic P16^INK4A^ driven approaches have been informative, but have caveats as described. Further use of inhibitors, and novel transgenic tools; for example, transgenic models allowing conditional induction of senescence in specific cell lineages at specific times in the presence/absence of tissue injury (Bird et al., 2018; Ferreira-Gonzalez et al., 2018) have the potential to enhance our understanding further. Such models should allow a deeper characterization of the pathways to senescence at single cell level; i.e., from “early” to “deep” senescence and permit further delineation of the effect of senescence on organ function and fibrosis in the absence of other general processes such as age or injury.

#### Can We Have the “Good” Features of Senescence Without the “Bad”?

As described, senescence is important for wound healing (Krizhanovsky et al., 2008; Jun and Lau, 2010; Demaria et al., 2014). With respect to renal injury, it is likely that optimal recovery requires the induction of senescent cells (most likely of different cell lineages) at key time-points. Thus, a delicate balance is required to promote adaptive rather than maladaptive repair. The specific identity and roles of these cells in renal repair versus development of fibrosis remain unknown at present and it is imperative that we understand this to deliver optimal senolytic therapies.

## Conclusions

Over the last five years depletion of senescent cells in animal models has demonstrated striking results across multiple tissues, organs and at whole animal level and this continues to inspire the development and refinement of therapy. However, as yet, their efficacy and safety has not been extensively tested in humans.

Multiple early stage clinical trials are underway and early reports are encouraging (Hickson et al., 2019; Justice et al., 2019). These include NCT02848131, a trial which tests dasatinib and quercetin as senolytics in diabetic patients with chronic kidney disease, NCT04063124 which will test the same agents in the modulation of progression of Alzheimer's disease and NCT04313634 comparing dasatinib, quercetin and fisetin on senescence levels and bone mineral density in elderly women. There are also trials on-going looking at the effects of known senolytics/senostatics on frailty and aging; in particular fisetin (NCT0367524) and metformin (NCT02915198, NCT03451006) whose results will clearly be of interest to the field in general. Additionally, the mdm2/p53 inhibiting peptide UBX0101 (NCT04129944) is under ongoing investigation as a local treatment for symptomatic osteoarthritis of the knee.

An important question relates to how the essential roles and/or beneficial effects of senescence in development, tissue repair and the opposition of malignancy can be retained while their negating their apparently deleterious actions on tissue fibrosis and dysfunction. It is to be hoped that in the next five years many of these questions will be answered. Current and future studies will undoubtedly broaden our knowledge of the key pathogenic mechanisms of senescent cells and hence permit refined therapies to deplete senescent cells in a targeted manner. It may also be possible to target the metabolic alterations present with senescence to deliver selective therapies to limit senescent cell survival. Finally, the presence of clinically licenced compounds with the ability to restrain the deleterious effects of senescence *via* inhibition of SASP release may allow these to be the first agents to transition from experimental models to clinical use.

## Author Contributions

MD planned the study and wrote and revised the text. DB planned the study and wrote the text. JH reviewed and revised the text. DF planned the study, designed the figures, and reviewed and revised the text.

## Funding

MD is funded by an MRC Clinical Research Training Fellowship. DF is supported by grant funding from Kidney Research UK (KRUK) and The Chief Scientist Office (CSO) Scotland.

## Conflict of Interest

The authors declare that the research was conducted in the absence of any commercial or financial relationships that could be construed as a potential conflict of interest.
